# Lung Cancer Adverse Events Reports for Angiotensin-Converting Enzyme Inhibitors: Data Mining of the FDA Adverse Event Reporting System Database

**DOI:** 10.3389/fmed.2021.594043

**Published:** 2021-02-01

**Authors:** Long Meng, Bing Yang, Feng Qiu, Yuntao Jia, Shusen Sun, JunQing Yang, Jing Huang

**Affiliations:** ^1^Department of Pharmacy, The First Affiliated Hospital of Chongqing Medical University, Chongqing, China; ^2^Nursing College, Chongqing Medical University, Chongqing, China; ^3^Ministry of Education Key Laboratory of Child Development and Disorders, Chongqing Key Laboratory of Pediatrics, Department of Pharmacy, China International Science and Technology Cooperation Base of Child Development and Critical Disorders, Children's Hospital of Chongqing Medical University, Chongqing, China; ^4^Department of Pharmacy Practice, College of Pharmacy and Health Sciences, Western New England University, Springfield, MA, United States; ^5^Department of Pharmacy, Xiangya Hospital Central South University, Changsha, China; ^6^Department of Pharmacology, Chongqing Medical University, Chongqing, China; ^7^Department of Respiratory and Critical Care Medicine, The First Affiliated Hospital of Chongqing Medical University, Chongqing, China

**Keywords:** angiotensin-converting enzyme inhibitors, lung cancer, pharmacovigilance, data mining, FAERS

## Abstract

Because of contradictory evidence from clinical trials, the association between angiotensin-converting enzyme inhibitors (ACEIs) and lung cancer needs further evaluation. As such, the current study is to assess disproportionate reporting of primary malignant lung cancer among reports for ACEIs submitted to the FDA adverse event reporting system utilizing a pharmacovigilance approach. We conducted a disproportionality analysis of primary malignant lung cancer adverse events associated with 10 ACEIs by calculating the reported odds ratios (ROR) and information component (IC) with 95% confidence intervals (CI). ROR was adjusted for sex, age, and reporting year by logistic regression analyses. From January 2004 to March 2020, a total of 622 cases of lung cancer adverse event reports were identified for ACEIs users. Significant disproportionate association was found for ACEIs as a drug class (ROR: 1.22, 95% CI: 1.13–1.32; IC: 0.28, 95% CI: 0.17–0.39. adjusted ROR: 1.23, 95% CI: 1.02–1.49). After stratification based on gender, a subset analysis suggested that female patients exhibited a significant disproportionate association, while male patients did not. Sensitivity analyses that limited the data by reporting region, comorbidity, and reporting year also showed similar trends. Statistical significant lung cancer signals were detected among patients who received ACEI, especially female patients. The disproportionality analysis of the FAERS database suggests mildly increased reporting of lung cancer among ACEI users. Further robust epidemiological studies are necessary to confirm this relationship.

## Background

Angiotensin-converting enzyme inhibitors (ACEIs) are one of the most widely prescribed antihypertensive medications. ACEIs are also used in the treatment of heart disease, diabetic nephropathy, and chronic kidney disease. ACEIs display their mechanism of action by inhibiting the renin-angiotensin-aldosterone system (RAAS) by decreasing the formation of angiotensin II (1). Alternatively, biological studies found angiotensin II acts on the AT1 receptor to promote cell proliferation and angiogenesis. These actions may make crucial implications for cancer development (2, 3).

Considering that hypertensive patients need lifelong therapy, concerns have been put forward that long term use of ACEIs may be associated with an increased risk of cancer (4). However, the carcinogenic potential of ACEIs has been subject to debate, especially in lung cancer. Multiple observational studies exhibited mixed results, including the increased, decreased, or unchanged risk of lung cancer in patients treated with ACEIs (5, 6). Based on a recent cohort of 992,061 patients treated with antihypertensive drugs, Hicks et al. reported that the use of ACEIs was associated with a 1.14-fold higher risk of lung cancer than the use of angiotensin receptor blockers (ARBs) (7). However, in Assimes's study, there was no significant association between ACEI use and the lung cancer development. Given the contradictory evidence from clinical trials, there is a need to evaluate the link between ACEIs and lung cancer from a new perspective.

Data mining of adverse event spontaneous reporting system (SRS) has been carried out to assess safety reflecting drug utilization in clinical practice. The U.S. Food and Drug Administration (FDA) adverse event reporting system (FAERS) provides information on medication error reports and adverse events (AEs). Data mining algorithms are routinely applied for the quantitative detection of signals, such as drug-associated AEs (8). Therefore, we aim to assess the potential relevance between ACEIs and primary malignant lung cancer AE reports through data mining of the FAERS.

## Methods

### Data Source

To identify lung cancer AE disproportionally reported following the use of ACEIs, a case/non-case study was conducted using spontaneous reports submitted in FAERS between the first quarter (Q1) of 2004 and the Q1 of 2020. All data from the SRS database were fully anonymized by the regulatory authorities before being used in the analysis.

OpenVigil FDA, a validated pharmacovigilance tool, was adapted to request FAERS data using the openFDA application programming interface to access the FDA drug-event database with the additional openFDA drug mapping and duplicate detection functionality (9, 10), and it is used in many pharmacovigilance studies (11, 12). OpenVigil operates only on cleaned FDA data by deleting most of the duplicates or reports with missing data (9). After data cleaning by OpenVigil FDA, 7,861,515 reports from 2004 Q1 to 2020 Q1 remained for data analysis.

### Definition of Adverse Events

Adverse events in FAERS reports are coded using the Medical Dictionary for Regulatory Activities (MedDRA, version 19.0) of Preferred Terms (PTs). PTs are intended to represent a single medical concept and linked with broader Higher Level Terms (HLT), Higher Level Group Terms, and System Organ Classes.

In our study, cases were represented by the reports retrieved under the MedDRA HLT term “Lower respiratory tract neoplasms” for any FDA-approved ACEI (benazepril, captopril, enalapril, fosinopril, lisinopril, moexipril, perindopril, quinapril, ramipril, and trandolapril) as suspected, interacting or concomitant, and irbesartan was chosen as the control drug. Several PTs (carcinoid tumor pulmonary, leukaemic infiltration pulmonary, endobronchial lipoma, benign respiratory tract neoplasm, benign lung neoplasm and metastases to lung) that were subordinated to the HLT “Lower respiratory tract neoplasms” were excluded because these terms are not primary malignant lung cancer. Non-cases were defined as all other reports. Non-FDA approved ACEIs (e.g., cilazapril and imidapril) were not included because they would suffer from underreporting rates in FAERS.

### Data Mining Algorithm

As measures of disproportionality (known as a case/non-case method), the reporting odds ratio (ROR) and information component (IC), along with a 95% confidence interval (CI), were calculated to identify drug-associated adverse events as signals (13, 14). ROR is frequentist (non-Bayesian), whereas the IC is Bayesian. ROR and IC are recognized disproportionality methods to identify whether a given AE (in this case, lung cancer) is reported more frequently than expected with a given drug (in this case, ACEIs), which allows testing the possible disproportionate association between a drug and an adverse event. The ROR is the odds of a specific AE occurring in a patient exposed to a drug of interest divided by the odds of an AE specific to another drug. The IC is a logarithmic metric of the value, which is calculated by dividing the likelihood of drug use and a specific AE by the product of the probability of drug use and the probability of a particular AE occurring when drug use and specific AE occurring are independent (15). The equations and criteria for the two algorithms (14, 16) are shown in Supplementary Table 1. Signals for AE were detected when at least one of two indices met the criteria. The WHO definition of a signal is “reported information on a possible causal relationship between an adverse event and a drug” (17). Basically, the higher the ROR or IC score, the stronger the disproportion appears to be (13). The ROR allows for adjustment using logistic regression analysis (18, 19) and has the advantage of controlling the following covariates: sex, age, and reporting year. The results were expressed as adjusted ROR (aROR). Reports with missing values for the covariates mentioned above were excluded. Thiazides, which did not show increased and decreased risk of lung cancer occurrence from a previous study (20), were used as the reference group.

Also, a gender subset analysis was performed to further demonstrate whether gender influences the reporting of lung cancer. As a sensitivity analysis, we recalculated the data mining statistics of ACEI as a class by (a) removing AEs from Europe, (b) removing AEs of non-small lung cancer subjects, and (c) restricting to subjects with diabetes to check for a potential source of bias. An additional sensitivity analysis with the timeframe from 2004 to 2011 was conducted to determine if the disproportionate reporting of lung cancer in patients using ACEIs might have been under- or over-estimated by published large scale clinical trials and meta-analyses, which have suggested increased and decreased risk of ACEIs and lung cancer occurrence (20, 21).

## Results

Overall, 197,320 AE reports related to ACEIs and 20,403 AE reports on lung cancer were submitted to the FAERS in the study period. Of these, a total of 11,248 AE reports were found in reports pertaining to benazepril, 4,316 for captopril, 22,179 for enalapril, 3,088 for fosinopril, 98,268 for lisinopril, 427 for moexipril, 9,383 for perindopril, 7,055 for quinapril, 41,214 for ramipril, and 2,221 for trandolapril.

The characteristics of AE reports submitted for ACEIs are described in Table 1. The gender subset analysis showed that ACEI reports associated with lung cancer were higher in female patients than male patients (50.2 vs. 46.0%; in 3.8%, the sex of the involved patient was unknown or missing). The largest percentages of reports (28.0%) were in patients aged 45–64 years. Cases exposed to ACEIs were mainly from the United States (67.5%), Canada (5.3%), and the United Kingdom (4.2%). ACEIs is most frequently used for unknown indication (*n* = 465), hypertension (*n* = 167) and heart disease (*n* = 9).

**Table 1:** The characteristics of adverse events reports of ACEIs.

**Characteristics**	**Cases^a^ (%)**	**Non-cases^b^ (%)**
**Patient gender**		
Male	286(46.0%)	90,178(45.7%)
Female	312(50.2%)	90,648(45.9%)
Unknown or missing	24(3.8%)	16,494(8.4%)
**Patient age group (years)**		
<18	1(0.2%)	1,493(0.8%)
18–44	8(1.3%)	10,579(5.4%)
45–64	174(28.0%)	55,526(28.1%)
65–74	143(23.0%)	37,965(19.2%)
>75	63(10.1%)	35,260(17.9%)
Unknown or missing	233(37.4%)	56,497(28.6%)
**Reporting country**		
United States	420(67.5%)	116,190(58.9%)
Canada	33(5.3%)	6,227(3.2%)
United Kingdom	26(4.2%)	21,265(10.7%)
Germany	21(3.4%)	9,835(5.0%)
Other countries	71(11.4%)	33,820(17.1%)
Unknown or missing	51(8.2%)	9,983(5.1%)
**Reporting region**		
America	464(74.6%)	125,328(63.5%)
Europe	96(15.4%)	56,737(28.8%)
Asia	6(1.0%)	3,054(1.5%)
Oceania	4(0.6%)	1,673(0.8%)
Africa	1(0.2%)	545(0.3%)
Unknown or missing	51(8.2%)	9,983(5.1%)
**Serious outcome of adverse events**		
Hospitalization (initial or prolonged)	323(51.9%)	75,116(38.1%)
Disability	27(4.3%)	5,763(2.9%)
Life-threatening	52(8.4%)	11,266(5.7%)
Death	181(29.1%)	15,805(8.0%)

a *Number of patients with primary malignant lung cancer adverse events*.

b *Number of patients without primary malignant lung cancer adverse events*.

**Figure 1:**
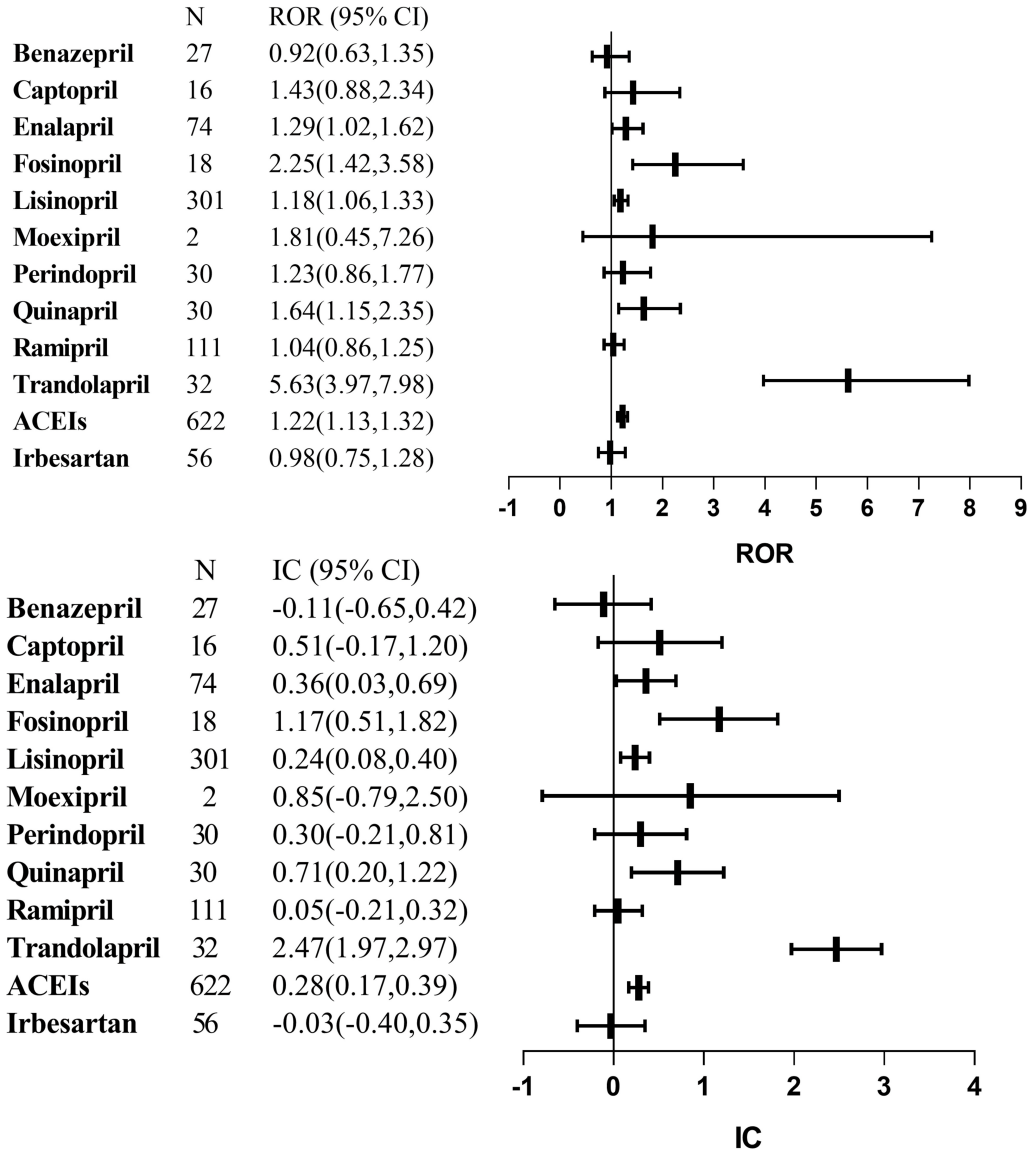
Signal detections for angiotensin-converting enzyme inhibitors-associated lung cancer. ACEIs, angiotensin-converting enzyme inhibitors; CI, confidence interval; IC, information component; ROR, reporting odds ratio.

Figure 1 lists the results of disproportionality analysis between ACEIs and lung cancer. Overall, based on the criteria for the two algorithms, the signal of lung cancer was detected for ACEI assessed together as a drug class (ROR: 1.22, 95% CI: 1.13–1.32; IC: 0.28, 95% CI: 0.17–0.39). After adjusting sex, age, and reporting year, aROR for the ACEI class was 1.23 (95% CI, 1.02–1.49).

As a single agent, we found statistically significant lung cancer signals for the following agents: enalapril, fosinopril, lisinopril, quinapril, and trandolapril. Benazepril, captopril, moexipril, perindopril, and ramipril were not identified.

**Figure 2:**
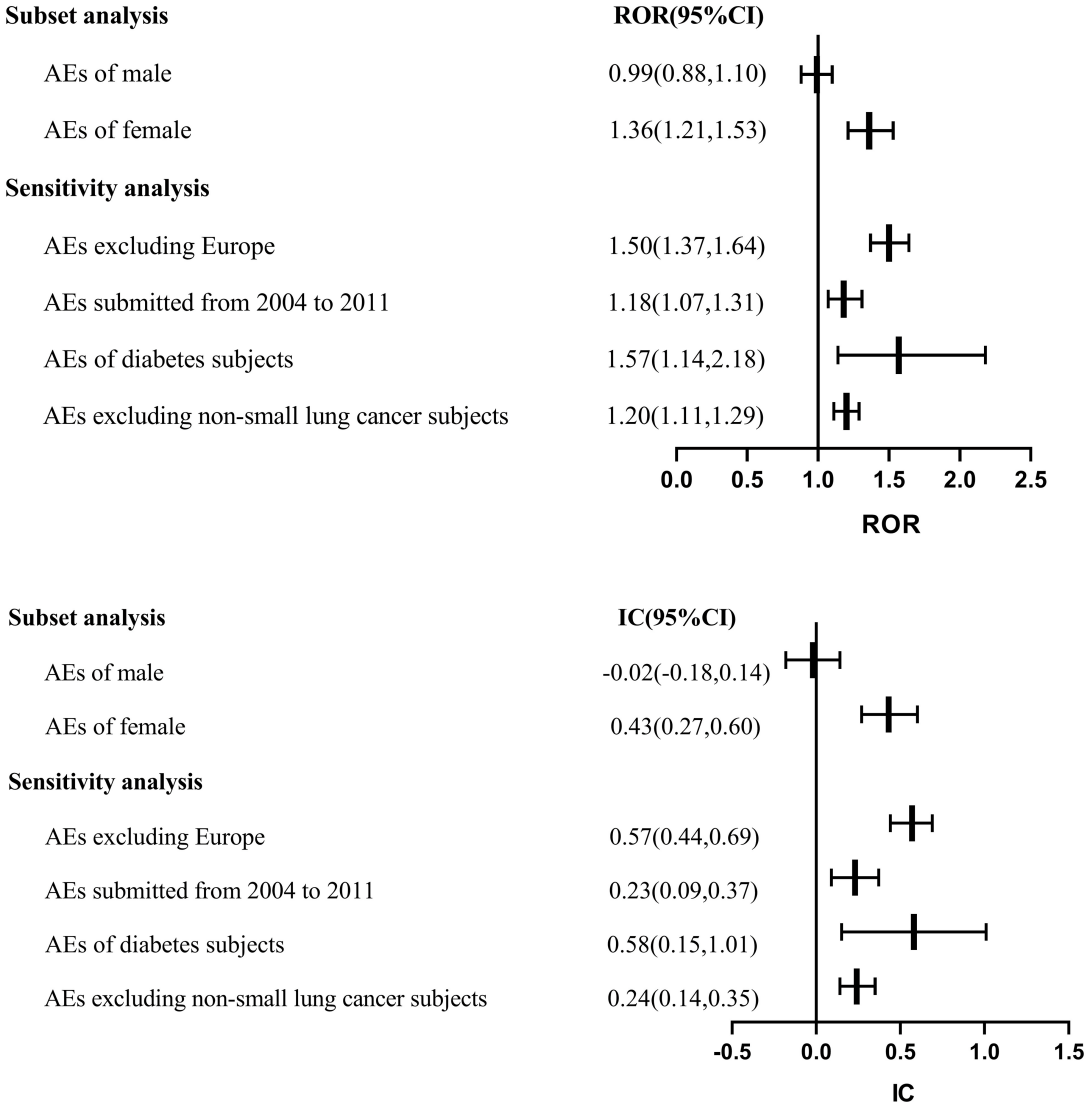
Subset and sensitivity analyses. AE, adverse event; CI, confidence interval; IC, information component; ROR, reporting odds ratio.

With regards to the gender subset, a significant signal of ACEI as a drug class was showed in female patients (ROR: 1.36, 95% CI: 1.21–1.53; IC: 0.43, 95% CI: 0.27–0.60) but not in male patients (ROR: 0.99, 95% CI: 0.88–1.10; IC: −0.02, 95% CI: −0.18 to 0.14) (Figure 2).

To test the robustness of the above findings, sensitivity analyses that limited (a) the submitted year of AE (ROR: 1.18, 95% CI: 1.07–1.31; IC: 0.23, 95% CI: 0.09–0.37), (b) AEs excluding non-small lung cancer subjects (ROR: 1.20, 95% CI: 1.11–1.29; IC: 0.24, 95% CI: 0.14–0.35), and (c) subjects with diabetes (ROR: 1.57, 95% CI: 1.14–2.18; IC: 0.58, 95% CI: 0.15–1.01) did not affect the results. Another sensitivity analysis removing AEs from Europe also showed a similar trend for ACEIs, consistent with the estimation of our primary analysis (ROR: 1.50, 95% CI: 1.37–1.64; IC: 0.57, 95% CI: 0.44–0.69) (Figure 2).

## Discussion

This study is the first analysis to investigate the potential link between ACEIs and primary malignant lung cancer using a pharmacovigilance approach. There is a disproportionate association of lung cancer among ACEIs users, especially in the female group based on our analysis. Undoubtedly, current literature reveals an inconsistent conclusion of the association between ACEIs and lung cancer. In Gokhale's study, it appeared that there was no evidence of an association between ACEIs and lung cancer incidence (hazard ratio = 0.99, 95% CI: 0.84–1.16) (22). Meta-analyses of randomized controlled trials found no risk of lung cancer and even decreased risk in patients taking ACEIs (23, 24). On the other hand, a meta-analysis with 324,168 patients from randomized trials demonstrated that a combination of an ACEI and an ARB significantly increased the risk of cancer (4). In another study, the increased risk of lung cancer was observed in the patients who received ACEIs (relative risk 1.13; 95% CI: 1.06–1.20) (25). According to a cohort study that included 992,061 participants who took antihypertensive drugs in the UK, the use of ACEIs was associated with an increased risk of lung cancer (incidence rate of 1.6/1,000 person-years; hazard ratio 1.14, 95% CI: 1.01–1.29). The correlation manifested stronger among patients taking ACEIs for more than 5 years in further analysis (7). Our study results are in accord with these meta-analyses and observational studies, although the absolute risk increase is modest.

Sensitivity analysis indicated the robustness of our results, conducted by restricting to specific values: subjects without non-small lung cancer, subjects with diabetes, and the years and region. Epidemiologic evidence shows that diabetes is strongly associated with cancer incidence (26). As one of the representatives' of ARBs, irbesartan was chosen as the control drug. The results demonstrated no significant lung cancer signals in the irbesartan group (ROR: 0.98, 95% CI: 0.75–1.28; IC: −0.03, 95% CI: −0.40 to 0.35), similar with the previous research (24). Although the potential mechanism of ACEI-induced lung cancer is unclear; several plausible possibilities have been raised. The use of ACEIs results in the accumulation of bradykinin in the lungs. Bradykinin, as an active vasodilator, has the potential to stimulate the growth of cancer cells through binding with various receptors (27). It was found that bradykinin induces tumor-associated angiogenesis by promoting the release of vascular endothelial growth factor (VEGF), facilitated cancer invasion, and metastases by activating matrix metalloproteinase (28, 29). The recent study indicated bradykinin promoted inflammatory factor (interleukin-8, interleukin-6, and cyclooxygenase-2) secretion, thereby contributing to malignancy progression (30–32).

Furthermore, ACEIs could lead to the accumulation of substance P, which is involved in tumor proliferation, migration, and angiogenesis (33, 34). Our study indicated a significant signal of lung cancer in female patients taking ACEIs, but not in male patients, which supports this hypothesis. A dry cough is one of the most common adverse reactions with ACEI use, which is more common in women than in men (35, 36). The underlying mechanism of ACEI cough is related to the accumulation of bradykinin and substance P, which stimulate vagal afferent fibers and sub-serve the cough reflex (36–39). Polymorphism in the ACE gene has been suggested to be associated with the susceptibility to cough in women (40). Whether these mechanisms lead to different signals dependent on sex is unknown. Further studies are needed to confirm this association and explore the mechanism.

It is noteworthy that hypertensive patients appears to have a higher risk of developing cancer, including lung cancer (41, 42). There's a trend to develop cancer and hypertension with aging. The increased levels of VEGF play a crucial role in tumorigenesis (43).

The data mining of the FAERS database is considered a valuable tool; however, this study has several limitations. First, this study fails to evaluate the causal relationship. Due to some inherent limitations of SRSs (44), it is a reasonably descriptive study applying the data-mining technique to identify potential significant drug/event combinations highlighting combinations that need further clinical validation. Second, the incompleteness of data in the FAERS dataset does not allow for extensive analysis of the potential effect of demographics, duration of use, and dosage strengths that might affect the association between ACEIs and lung cancer. Third, a detection bias may exist because patients with intolerable cough due to ACEIs may receive more chest examinations, leading to an increased probability of an early diagnosis of lung cancer. Therefore, causality cannot be confirmed based on the FAERS data alone. Notwithstanding these limitations, our analysis has important strengths. First, our study is the first to attempt to investigate the potential link between the use of ACEI and lung cancer by using the FAERS database. FAERS is the largest publicly available SRS, which contains data of unselected real-world patients globally, which has been collected for decades. Second, the study offers a unique opportunity to detect and reevaluate, in a timely and inexpensive manner, the risk-benefit profile of drugs, which is different from clinical trials to assess drug safety. Third, to minimize the reporting bias, sensitive analysis restricting the reporting region, comorbidity, and reporting year was conducted. We performed both the ROR and IC algorithms in the FAERS database analysis and detected reliable signals.

Because of contradictory evidence from clinical trials, the association between ACEIs and lung cancer remains unclear. In the present study, the debate was further investigated by new insights gained from pharmacovigilance. While pharmacovigilance studies using the FAERS database have limitations, as mentioned above, they can identify signals between ACEI regimens and lung cancer, which provides clues for pharmacoepidemiological studies for further evaluating the causal relationship between a signal and ACEI-associated lung cancer.

## Conclusions

Our analysis finds an association between ACEIs and primary malignant lung cancer risk. The risk is higher in female patients than in male patients.

## Data Availability Statement

The raw data supporting the conclusions of this article will be made available by the authors, without undue reservation.

## Author Contributions

LM took primary responsibility for conducting this study. JH and LM drafted the manuscript with support from SS and YJ. All authors contributed to conception and study design, and participated in data collection, analyses, and interpretation, and revisions of the manuscript and approved the final version.

## Conflict of Interest

The authors declare that the research was conducted in the absence of any commercial or financial relationships that could be construed as a potential conflict of interest.
