# Supplementary material for: Lung Cancer Adverse Events Reports for Angiotensin-Converting Enzyme Inhibitors: Data Mining of the FDA Adverse Event Reporting System Database
Source: Front Med (Lausanne). 2021 Feb 1;8:594043. doi: 10.3389/fmed.2021.594043 (PMC7882608; doi:10.3389/fmed.2021.594043)
Supplement: Supplementary file 1 [file Table_1.docx]

Supplementary Material Table 1**.** Summary of the algorithms used for signal detection.

| Algorithms | Equation | Criteria |
| --- | --- | --- |
| ROR | ROR=(ad)/(cb)  95% CI=$e^{\ln\left( ROR \right)\pm1.96\sqrt{1/a+1/b+1/c+1/d}}$ | 95% CI > 1, a > 2 |
| BCPNN | IC=$\log_{2} a(a+b+c+d)(a+c)(a+b)$  95% CI=$e^{\ln\left( IC \right)\pm1.96\sqrt{1/a+1/b+1/c+1/d}}$ | IC025 > 0 |

Abbreviations: a: the number of reports with interest adverse event of the suspect drug; b: the number of reports with the interest adverse event of all other drugs; c: the number of reports with all other adverse events of the suspect drug; d: the number of reports with all other adverse events of all other drugs; ROR: reporting odds ratio; BCPNN: Bayesian confidence interval progressive neural network; CI: confidence interval; IC: information component; IC025: the lower limit of the 95% two-sided confidence interval of the information component.
